# Securin overexpression correlates with the activated Rb/E2F1 pathway and histone H3 epigenetic modifications in raw areca nut-induced carcinogenesis in mice

**DOI:** 10.1186/s12935-022-02442-z

**Published:** 2022-01-15

**Authors:** Nabamita Boruah, Chongtham Sovachandra Singh, Pooja Swargiary, Hughbert Dkhar, Anupam Chatterjee

**Affiliations:** 1 Histopathology Division, Nazareth Hospital, Laitumkhrah, Shillong, 793003 India; 2 Molecular Genetics Laboratory, Department of Biotechnology & Bioinformatics, North-Eastern Hill University, Shillong, Meghalaya 793022 India

**Keywords:** PTTG1/Securin gene, Histone methylation, Histone acetylation, Rb phosphorylation

## Abstract

**Background:**

Raw areca nut (RAN) consumption induces oral, esophageal and gastric cancers, which are significantly associated with the overexpression of pituitary tumor transforming gene 1/securin and chromosomal instability (CIN). An association of Securin/PTTG1 upregulation and gastric cancer in human was also demonstrated earlier. Since the molecular mechanism underlying securin upregulation remains unclear, this study intended to investigate the association of securin upregulation with the Rb-E2F1 circuit and epigenetic histone (H3) modification patterns both globally and in the promoter region of the securin gene.

**Methods:**

Six groups of mice were used, and in the treated group, each mouse consumed 1 mg of RAN extract with lime per day ad libitum in the drinking water for 60 days, after which the dose was increased by 1 mg every 60 days. Histopathological evaluation of stomach tissues was performed and securin expression was analysed by immunoblotting as well as by immunohistochemistry. ChIP-qPCR assays were performed to evaluate the recruitment of different histone modifications in the core promoter region of securin gene as well as its upstream and downstream regions.

**Results:**

All mice developed gastric cancer with securin overexpression after 300 days of feeding. Immunohistochemistry data revealed hyperphosphorylation of Rb and upregulation of E2F1 in the RAN-treated samples. Increased trimethylation of H3 lysine 4 and acetylation of H3 lysine 9 and 18 both globally and in the promoter region of the securin gene were observed by increasing the levels of lysine-N-methyltransferase 2A, lysine-acetyltransferase, EP-300 and PCAF after RAN treatment. ChIP-qPCR data revealed that the quantity of DNA fragments retrieved from the immunoprecipitated samples was maximum in the -83 to -192 region than further upstream and the downstream of the promoter for H3K4Me3, H3K9ac, H3K18ac and H3K9me3.

**Conclusions:**

RAN-mediated pRb-inactivation induced securin upregulation, a putative E2F1 target, by inducing misregulation in chromatin remodeling in its promoter region, which led to transcriptional activation and subsequent development of chromosomal instability. Therefore, present results have led to the hypothesis that RAN-induced changes in the epigenetic landscape, securin overexpression and subsequent elevation of chromosomal instability is probably byproducts of inactivation of the pRb pathway.

**Supplementary Information:**

The online version contains supplementary material available at 10.1186/s12935-022-02442-z.

## Introduction

Mammalian securin which was initially isolated from rat pituitary tumor cells as pituitary tumor transforming gene 1 (PTTG1) [1], is an oncogene and has been implicated in the development and progression of several malignancies [2]. It encodes a protein that prevents separin from promoting sister chromatid separation during mitosis [3]. This gene product is involved in cell cycle progression, p53-mediated apoptosis, transcription activator of several other oncogenes [4] and DNA repair [5]. It has been demonstrated that higher expression of securin, induction of precocious anaphase (premature separation of sister chromatids) and chromosomal instability have been associated with an increased risk of raw areca nut (RAN)-induced oral, esophageal and gastric cancers in both humans and mice [6, 7]. In fact, it has been proposed that these parameters can be considered screening markers for the identification of mitotic checkpoint defects during the early days of RAN exposure. Traditionally, people in the northeastern region of India consume betel quid consisting of RAN, lime and a small portion of betel leaves without tobacco. After chewing, they usually swallow the whole quid, which is responsible for not only the induction of oral cancer but also esophageal and gastric cancers. Higher DNA damage, p53 overexpression, greater delay in cell kinetics and lower GSH levels in peripheral blood lymphocytes have been demonstrated in heavy RAN chewers than in non-chewers [8]. Such observations prompted us to propose that in addition to cytogenetic parameters, the levels of endogenous GSH and p53 protein could act as effective biomarkers for RAN chewers [8]. The mutagenicity and genotoxicity of RAN-alkaloids has been demonstrated in several short-term assays, and it has been suggested that RAN should be considered a human carcinogen since it induces preneoplastic and neoplastic lesions in experimental animals [9].

Upregulation of securin and subsequent dysregulation of chromosome segregation leading to chromosomal instability have been observed not only in human cancer cell lines but also in a vast array of malignancies, including pituitary, colorectal, thyroid, lung, prostate, oral and esophageal squamous cell carcinoma [10–18]. Increased levels of securin are correlated with higher tumor grade, invasiveness and tumor vascularity [19]. Thus, it has been proposed that the securin level may be considered a molecular marker that can be a potential therapeutic target for many cancers [20]. However, it is interesting to note that despite its clinical relevance, the molecular mechanisms underlying securin abundance remain elusive.

In a separate study, it was demonstrated that RAN-alkaloids treated mouse spleen and bone marrow cells showed higher DNaseI fragmentation indicating a more relaxed chromatin structure which could be considered a causative factor for RAN-induced carcinogenesis [21]. Chromatin remodeling occurs through the posttranslational modifications of basic amino acid residues of histone tails, which either cause activation or repression of gene expression [22–24]. It is now well documented that the structure and integrity of the genome can be altered by disrupting this complex epigenetic control mechanism, which ultimately alters the expression of genes that are critically involved in tumorigenesis [23, 25]. Therefore, the present study is intended to evaluate the epigenetic histone modification patterns in the promoter region of the securin gene because such modifications do have profound effects on gene promoter activity [26, 27]. It has been observed that hPTG1 expression was reduced in bladder cancer cells after knockdown of E2F3 by measuring the expression of cDNA microarray analysis [28]. Furthermore, it was shown that hPTTG1 may act as a direct E2F1 target, and both were concordantly overexpressed in Rb^+/−^ murine pituitary tissues and human pituitary tumors [29]. Therefore, expression of E2F1 and Rb phosphorylation and their association with the securin overexpression in RAN exposed mouse stomach cells is worth investigating.

It has become increasingly clear that both environmental factors and lifestyle can promote a wide range of epigenetic modifications that are causally involved in cancer development and progression [30]. Therefore, the objective of this study was to analyse the status of RAN-induced E2F1 expression, pRb phosphorylation and posttranslational histone H3 modifications both at the global level and in the promoter region of the securin gene by immunohistochemistry and a standard chromatin immunoprecipitation-qPCR (ChIP-qPCR) protocol. Our data reveal that Rb inactivation releases E2F1 to induce PTTG1/securin expression and show its ability to exert broader effects on transcriptional control and chromatin structure through epigenetic histone modifications in RAN-induced gastric cancers.

## Materials and methods

### Preparation of extracts

RAN was ground into fine powder, and 100 g of the powder was extracted with 125 ml of distilled water and mixed thoroughly to give a smooth paste for preparation of an aqueous extract of RAN. After 24 h at 4 °C, the paste was stirred for 3 h at room temperature and the aqueous extract was collected by centrifugation. This extraction procedure was repeated once more by adding 125 ml of water to the residue. Both extracts were pooled, representing 100 g of RAN in 250 ml distilled water, filtered and frozen at − 80 °C. The filtrate was lyophilized in a Scanlaf Coolsafe Lyophilizer (Lynge, Denmark). The lyophilized mass was kept at 4 °C until use. The extract contained 0.9 g/100 g water-extractable material.

### Animals maintenance and treatment

Swiss albino mice (25–30 gm) aged 2–3 months were maintained in the laboratory in community cages and housed in the Animal Resource Facility of the university under the following conditions: 12 h dark/12-h light cycle, 20 ± 2 °C temperatures and 50 ± 10% humidity. A standard mouse diet (NMC Oil Mills Ltd., Pune, India) and water ad libitum were used in all experiments. A total of six groups of mice (n = 7 in each) were used at 0, 60, 100, 180, 240 and 300 days for different experimental analyses. One group was treated with simple drinking water, which was considered an untreated control, whereas the other five groups were administered RAN extract ad libitum in the drinking water with slaked lime (calcium hydroxide; pH 9.8). It was estimated that each mouse consumes 5 to 6 ml water in a day. Thus, RAN extract was mixed in the water in such a way that each mouse consumed 1 mg of extract per day. Such oral administration was continued for 60 days, after which the dose was increased from 1 to 2 mg per day until 120 days. Every 60 days later, the dose was increased by 1 mg per day consumption. In the present study, the mice were fed until 300 days. The dose and the treatment pattern were similar to our earlier study where it was shown that continuous ad libitum administration of RAN extract with lime in drinking water for 220 days or more can induce stomach and esophageal cancer in mice [6]. This study was carried out in strict accordance with the institutional guidelines (pls see ‘Ethics approval’). All animals were treated humanely, and they were euthanized by CO_2_ inhalation at their home cage (delivering 75% CO_2_ for a minimum of 3 min) followed by cervical dislocation as per required experimental time point.

### Histopathological evaluation

Stomach tissues of mice were collected from untreated and treated with RAN + lime for 300 days and preserved in 10% formalin. Three mice were selected from the untreated group, and none of them showed any indication of tumor externally. All seven mice in the treated group for 300 days were selected for histological evaluation. Tissues were processed for histological sectioning according to a standard protocol [31]. Formalin-fixed paraffin- embedded tissue blocks were serially sectioned (5 μm) with a microtome (Leica Biosystems, Wetzlar, Germany) and stained with hematoxylin and eosin [32]. Sections were then observed under a light microscope and photographed (Carl Zeiss, Oberkochen, Germany).

### Immunoblotting

Cells were collected from the inner layer of the stomach from untreated (n = 2) and RAN + lime- treated mice for 300 days (n = 3). The cells were washed with ice-cold 0.1 M phosphate-buffered saline (PBS; pH 7.4), and total protein was extracted with lysis buffer containing 0.1% SDS, 2 mM EDTA, 1% NP-40, 1% sodium deoxycholate, 50 mM sodium fluoride, 100 U/ml aprotinin and 1 mM phenylmethylsulfonyl fluoride. After centrifugation, the cell lysate was collected and the protein concentration was determined using the bicinchoninic acid protein assay. Equal amounts of protein (40 µg/well) were subjected to Novex Tris–Glycine 4–20% gradient gels, and electrophoresis was performed in a NuPAGE electrophoresis system (Invitrogen, California, USA). Then the proteins were transferred to a polyvinylidene difluoride membrane (Sigma) and probed with 1:1000 dilution of a mouse monoclonal antibody against securin (DCS-280; ab3305; Abcam, California, USA) and β-actin (AC-15; ab6276; Abcam, USA). Alkaline–phosphatase conjugated anti-mouse IgG (Abcam, USA) was used as the secondary antibody, and immunodetection was performed by treating the blot with the substrate solution of BCIP/NBT (Bangalore Genei, India).

### Immunohistochemistry (IHC) analysis

Stomach tissues of mice were collected from untreated and treated with RAN + lime and preserved in 10% formalin. Four mice were selected from each group. Tissue samples were dehydrated, paraffin embedded and sectioned with a microtome. Briefly, after blocking for endogenous peroxidase activity, the sections of stomach tissues were incubated with anti-securin (DCS-280; ab3305; Abcam, UK), anti-H3K4me3 (histone H3 lysine 4 trimethylation) primary antibody (ab8580; Abcam, UK), anti-H3K9me3 (histone H3 lysine 9 trimethylation) primary antibody (ab8898; Abcam, UK), anti-H3K9Ac (histone H3 lysine 9 acetylation) primary antibody (ab12179; Abcam, UK), anti-H3K18ac (H3 lysine 18 acetylation) primary antibody (ab1191; Abcam, USA), anti-Rb-phosphorylation primary antibody (SC-271930; Santa Cruz Biotechnology, USA), anti-E2F1 primary antibody (SC-22820; Santa Cruz Biotechnology, USA) and anti-KAT2A/GCN5 antibody (ab18381; Abcam, USA). IHC analysis was performed with a Strept-Avidin Biotin Kit (Dako, Agilent Technologies Company, Denmark). The scoring of immunohistochemical stains in each specimen was determined using a histological score (H) [33] (please see Additional file 1). Only Histone 3 antibody (ab1791; Abcam, UK) was used as an internal control.

### Chromatin immunoprecipitation assay (ChIP)

ChIP assays for the detection of posttranslational histone modification patterns in the upstream to downstream regions of the promoter of the securin gene were performed in mouse stomach cells. Stomach epithelial cells from four different animals at each point were lysed, sonicated and incubated with antibodies specific to H3K4me3 (ab8580, AbCam, UK), H3K9Ac (ab12179), H3K9me3 (ab8898), H3K18Ac (ab1191) and Histone 3 (ab1791) with protein A/G beads (Pierce™ Protein A/G Agarose, Cat no. 20421) incubated overnight at 4 °C. The methodology of ChIP is described in detail in the Additional file 1: Section.

Quantitative PCR (qPCR; BioRad CFX system) was used to quantitate amounts of DNA fragments in the immunoprecipitated samples from the ChIP analyses. qPCR was performed with reagents containing SYBR green and four specific primer sets located within + 626 bp to -874 bp of the promoter of PTTG1 in mice, as confirmed by sequencing (Science genome browser) of the qPCR products. qPCR was also performed in the immunoprecipitated samples from the ChIP analyses with a pair of primer sets representing the gene desert regions of chromosome 11 in mouse samples as a negative control. Samples were heated to 95 °C for 5 min and then amplified for 45 cycles at 95 °C for 30 s, 60 °C for 30 s and 72 °C for 30 s. Immunoprecipitated DNA was detected by qPCR and normalized to input DNA. Enrichment was calculated relative to input. qPCR products were purified using a SIGMA PCR clean up kit (NA 1020) and sent for sequencing to Agrigenome, Kochi, India.

### RNA extraction and qRT-PCR

qRT-PCR was performed to assess the transcriptional levels of KMT2A (lysine methyltr-ansferase 2A), KAT2A (lysine acetyltransferase 2A), EP300 (lysine acetyltransferase KAT3B), P300/CBP-associated factor (PCAF) also known as K(lysine) acetyltransferase 2B (KAT2B), HDAC3 (histone deacetylase 3), KDM4C (histone demethylase) and the reference gene GAPDH. Cell lysates were collected, and RNA was extracted and purified using a RNeasy® mini kit (Qiagen GmbH, Hilden, Germany) according to the manufacturer’s instructions. Reverse transcription of the RNA into cDNA was then performed using a QuantiTect Reverse Transcription kit (Qiagen GmbH, Hilden, Germany). qRT-PCR was performed in a Bio-Rad CFX96 Real-Time PCR Detection System using SYBR Green PCR Master Mix (Thermo Fischer Scientific, Massachusetts, United States). The primers used for qRT-PCR were listed in Additional file 1: Table S1.

### Statistical analysis

To compare the expression of securin between untreated and treated groups with different durations, unpaired Student’s t-test was performed for statistical analysis. The statistical significance of the levels of Histone3 K4-trimethylation, K9-acetylation, K9-trimethylation, K18-acetylation, pRb-phosphorylation, E2F1 activities and the expression of several epigenetic chromatin modification enzymes between the treated and untreated groups, was determined by one-way ANOVA. The Tukey test was used for post hoc analysis. The results are shown as the means ± SEM, and P < 0.05 was considered statistically significant. Statistical analysis was performed using GraphPad Prism 5.0.

## Results

### General observations

**Fig. 1:**
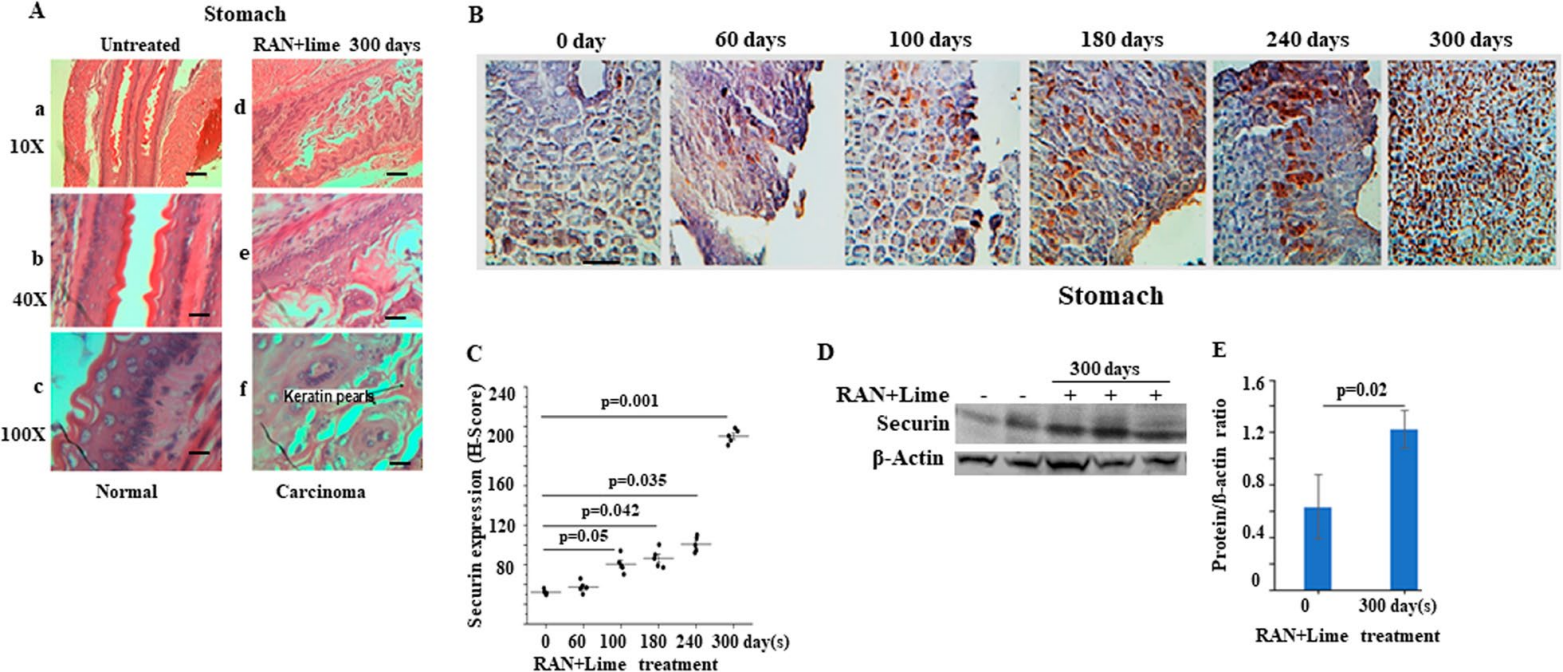
Expression analysis of the mouse PTTG1/securin gene in both normal and tumor tissues following treatment with RAN + lime.** A** Histopathology of untreated normal and tumorous stomach of mice following RAN + lime treatment for 300 days (scale bars: 200 µm). Carcinoma shows squamous keratin pearls. The magnification is indicated as 10X, 40X and 100X. **B** Immunohistochemical images of mouse stomach treated with RAN + lime for various durations. Normal expression of the securin gene in the untreated control and gradual upregulation of securin expression in treated samples for different time periods are shown. The magnification of all these images is × 40 and the scale bar: 200 µm. The expression level of securin in untreated and treated mice was analysed by H-score and is shown as the mean H-score ± SEM in (**C**). **D** Representative western blotting detection of securin and ß-actin in mouse stomach cells after exposure to RAN + lime for 0 and 300 days. **E** Quantitative densitometric analysis of the level of proteins of the genes mentioned in (**D**). The values are the mean ± SEM of the number of individuals used in this experiment. The values were normalized to the respective ß-actin values. In both **C** and **E**, the P values are shown compared with the untreated control (as determined by paired t-test). P values less than 0.05 are considered significant

In total, 42 mice, distributed in six groups, were used at different time points for different experimental analyses. After 300 days of feeding RAN extract with lime, all seven mice developed stomach cancer. Histological sections clearly differentiated between normal and tumorous stomachs (Fig. 1A, a–f) in mice.

### Securin overexpression through immunohistochemical staining

The expression of securin was evaluated by immunostaining in a panel of mouse stomach samples collected from untreated mice and treated with RAN + lime for 60, 100, 180, 240 and 300 days (Fig. 1B). The expression of securin increased gradually from 60 to 300 days of feeding with RAN + lime compared with the untreated control. At each point, five mice were used. The H-score in the untreated control was less than 40 and reached approximately 200 after 300 days of feeding (Fig. 1C).

### Overexpression of the securin gene through immunoblotting

The level of securin protein in stomach cells from mice after administration of RAN extract with lime for 0 and 300 days of feeding was examined by immunoblotting. The results indicated that the expression of the securin gene was elevated significantly after 300 days of feeding (Fig. 1D, E).

### Global methylation and acetylation pattern of histone H3 lysine 4, 9 and 18

**Fig. 2:**
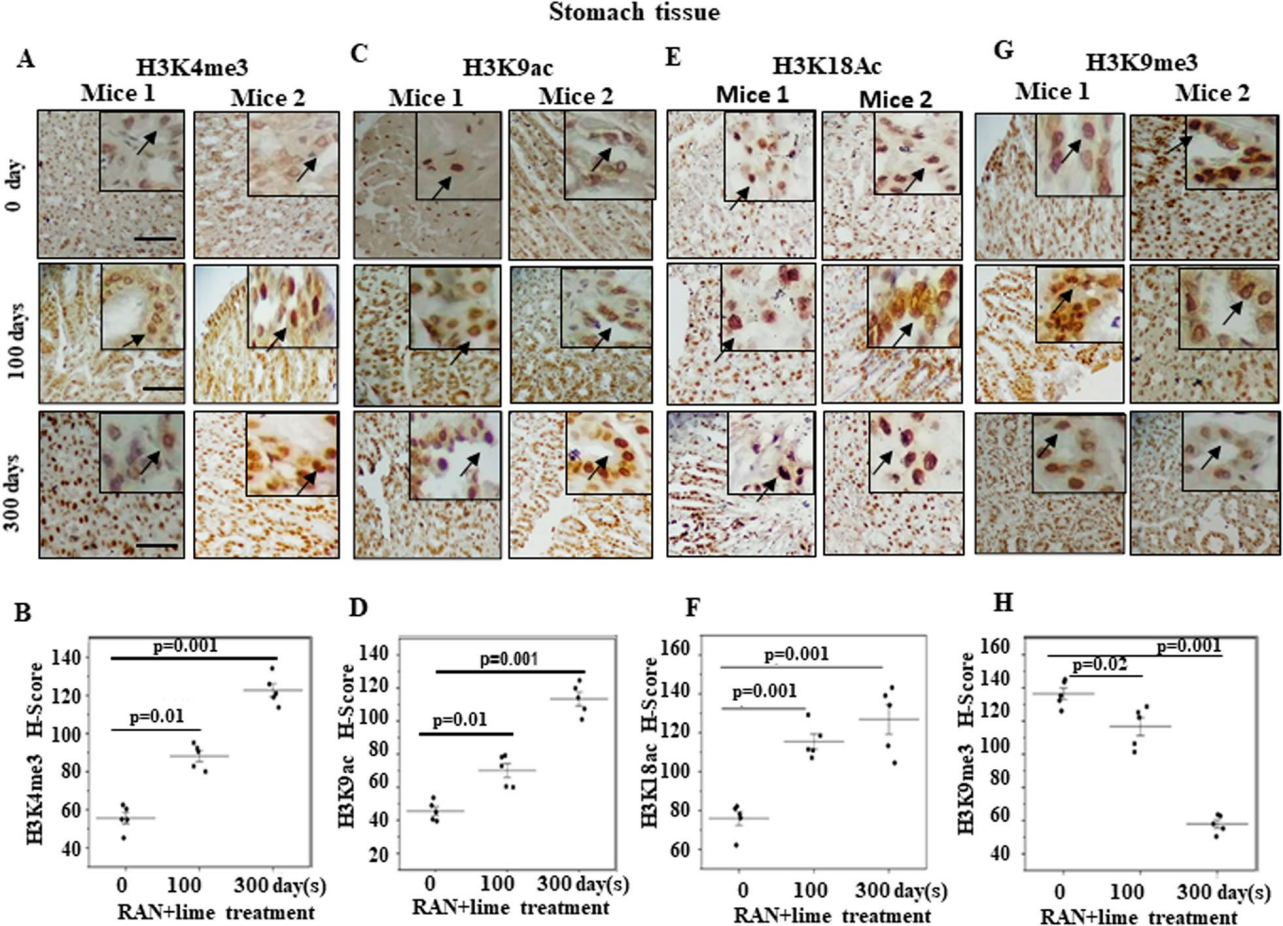
Immunohistochemical images of histone H3 methylation and acetylation in mouse stomach cells.** A** Representative images show that H3K4me3 positive cells increase with treatment duration. **B** Scatterplot of H-scores based on IHC for H3K4me3-positive cells. **C** Representative images show H3K9ac-positive cells increase with treatment duration. **D** Scatterplot of H-scores based on IHC for H3K9ac positive cells. **E** Representative images show that H3K18ac-positive cells increase with treatment duration. **F** Scatterplot H-scores based on IHC for H3K18ac-positive cells. **G** Representative images show that H3K9me3- positive cells decrease with treatment duration. **H** Scatterplot of H-scores based on IHC for H3K9me3-positive cells. The magnification of all these images is 40x. Inset: 100 × magnification as compared with adjacent cells. The arrow indicates nuclear localization. Data were analysed using one-way ANOVA with Tukey’s multiple comparison post-tests. In each case, images of two mice were shown. The scale bar: 200 µm

To examine the pattern of trimethylated and acetylated histone H3 at lysine 4, 9 and 18, we conducted immunohistochemistry on serial sections of mouse stomach (Fig. 2). The histo-chemical staining of H3K4me3, H3K9ac, H3K9me3 and H3K18ac appeared as brown particles and localized primarily within nuclei of stomach epithelial cells. As shown in Fig. 2A–F, the staining patterns of H3K4me3, H3K9ac and H3K18ac were very weak in untreated stomach samples and gradually stronger in 100- and 300-day treated samples. The mean H scores of H3K4me3, H3K9ac and H3K18ac were increased significantly after 100 and 300 days of RAN-treatment with respect to the untreated control. Interestingly, the staining pattern of H3K9me3 was reduced significantly after 100 and 300 days of RAN treatment with respect to the untreated control (Fig. 2G and H).

The IHC images with only Histone 3 antibody in stomach cells are shown to be similar in untreated and treated samples and served as an internal control (Additional file 1: Fig. S1 in the additional file section).

### Hyperphosphorylation of Rb and increased E2F1 expression after RAN treatment

**Fig. 3:**
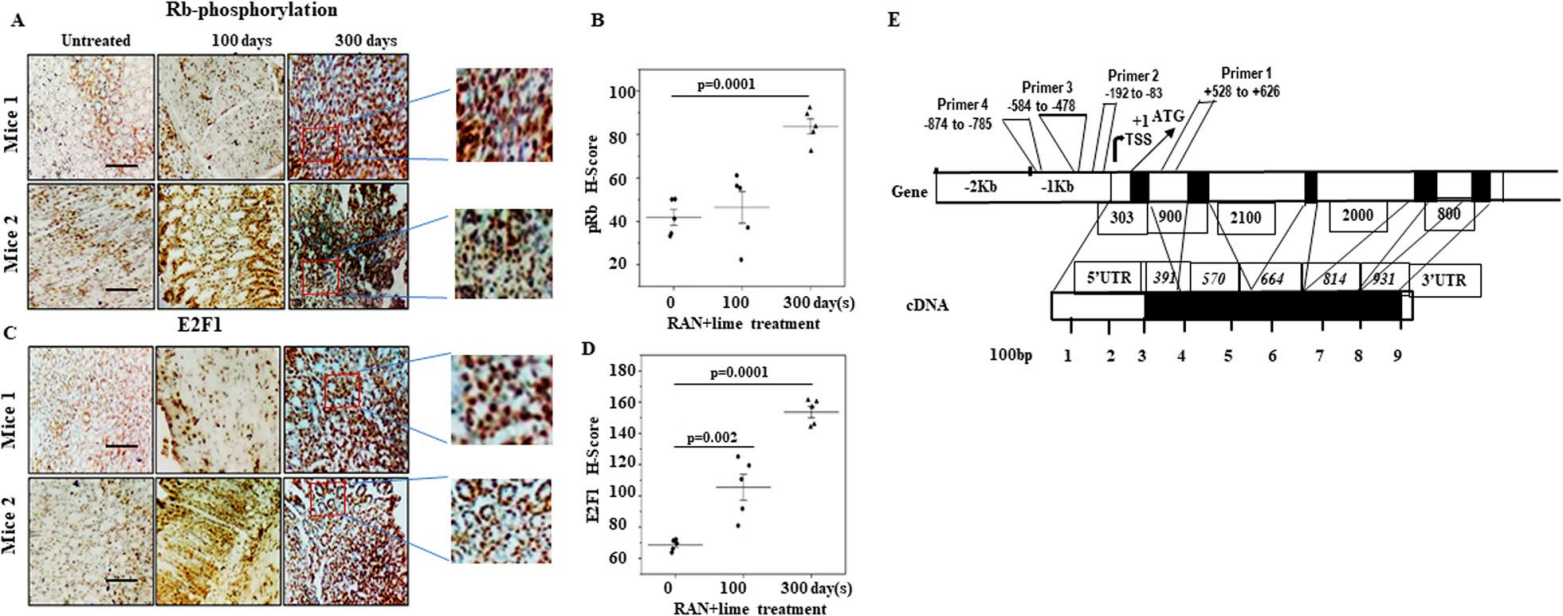
Immunohistochemical images for Rb phosphorylation and E2F1 expression and securin gene structure. Immunohistochemical assays of **A** phosphor-retinoblastoma (pRb) and **C** E2F1 expression in mouse stomach cells with and without RAN + lime treatment. Magnification (100x) of the marked area is shown. Grouped scatterplot illustrating quantitative values within each grouping of H-scores which are represented in **B** and **D** as the mean ± SEM; P-values were calculated with untreated control using one-way ANOVA with Tukey’s multiple comparison post-tests. In each case images of two mice were shown. The scale bar: 200 µm. **E** Schematic illustration of the mouse PTTG1/securin gene structure. Exons are indicated as boxes (translated sequences in black and untranslated regions in white). Exon and intron sizes (bp) are indicated. The transcription initiation site (TSS) is shown as + 1 and the translation start site ATG is indicated with an arrow. The promoter region is shown in Kb and the region is highlighted from where the primer pair was designed. The lower panel represents cDNA with a scale multiple of 100 bp. Both 5`- and 3`-flanking untranslated regions are shown

Immunohistochemical assays of pRb and E2F1 expression in mouse stomach cells with and without RAN treatment are shown in Fig. 3A–D. Hyperphosphorylation of RB and consecutive stimulation of E2F1 were clear in the RAN-treated samples.

### Securin gene structure in mice and primer design

The entire mouse PTTG1/securin gene spans approximately 7 kb and is composed of five exons and four introns. The exons were approximately about 391, 179, 94, 150, and 131 bp in size, and the four introns were approximately 0.9, 2.1, 2.0 and 0.8 Kb, respectively (Fig. 3E) [34]. The translation start site (ATG) is located 303 bp downstream of the single transcription start site, and a deletion scanning study of an active 4.3 kb upstream region revealed that sequences − 313 to − 150 bp are critical for promoter activity. Therefore, in total four primers, covering the upstream (− 874 bp) to the downstream (+ 626 bp) of the securin promoter region, were designed from the UCSC browser and with the help of Primer 3 software (Version 0.4.0). Usually, the core enhancer sequence is present in the upstream region of the securin gene promoter in both rats and humans and is critical for securin transcriptional activation [35, 36].

Primer details are given below:

Primer 1: Forward 5’-CGTCCTCAATGCCAATATCC -3’

Reverse 5’-AGGATAATGAAGAACCCGGC -3’

Primer 2: Forward 5’-GCCTCTCACAGGAGTTTTGG-3’.

Reverse 5’-CGCGTCTGTCTCCAAAGTATT-3’.

Primer 3: Forward 5’-GGCCTGTTCCCCTAGAGATT-3’.

Reverse 5’- CAGGCTTTTCGGAAACTCAC-3’

Primer 4: Forward 5’-CAGGCCATCCTGGTCTACAT-3’.

Reverse 5’-GGAGAGATTCCTGGGCAGTT-3’.

A pair of primer sets representing the gene desert regions of chromosome 11 in mouse was used in this study as a negative control. The primer sequences were taken from the previously published manuscript [37].

### ChIP analysis of chromatin composition

**Fig. 4:**
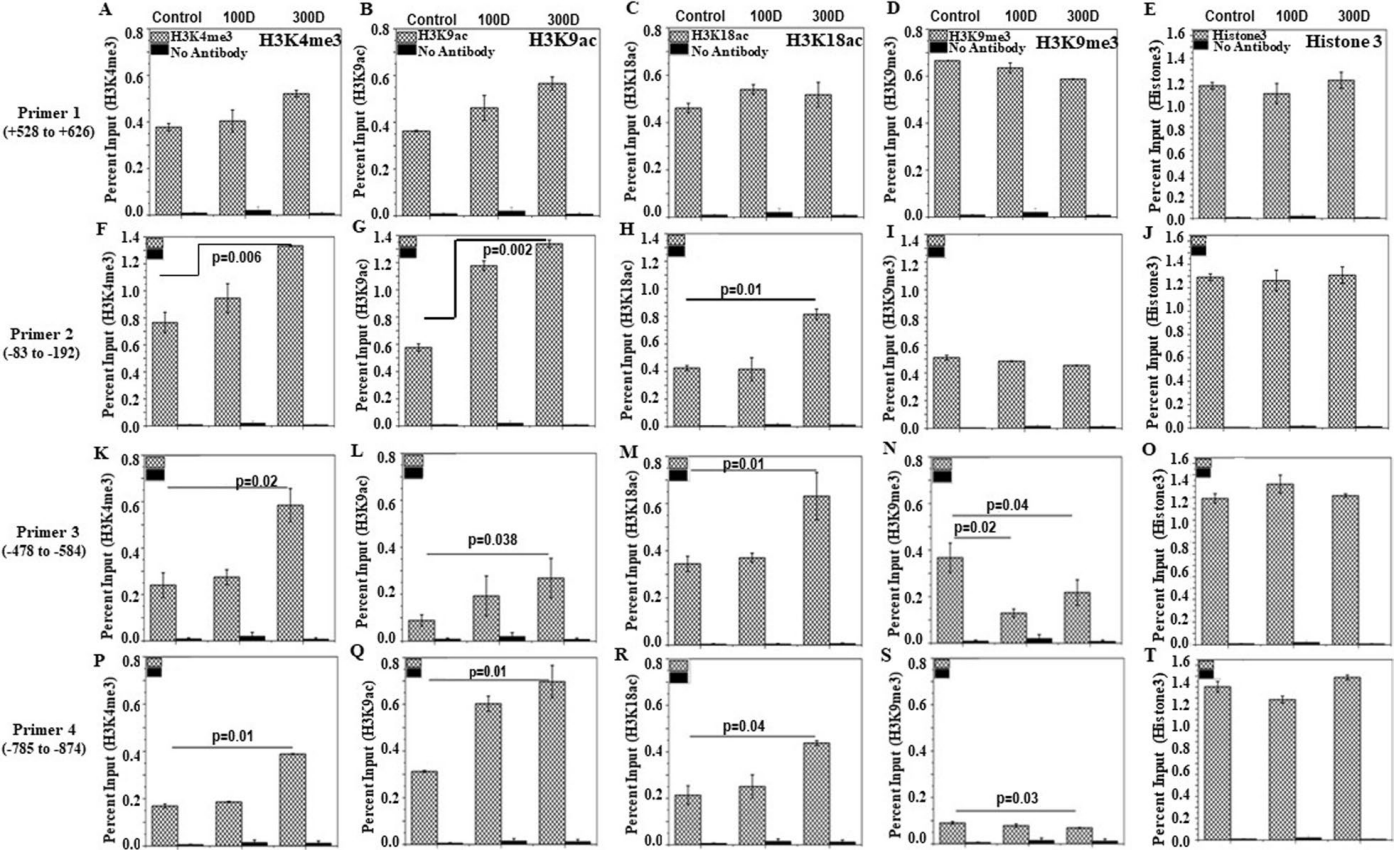
ChIP analysis of histone H3 methylation and acetylation at the region covering upstream to downstream of the promoter of the securin gene. ChIP-qRT-PCR assays for H3K4me3, H3K9ac, H3K18ac and H3K9me3 recruitment to the four regions of the securin gene + 528 to + 626 (upper panel **A**–**E**), − 83 to − 192 (**F**–**J**), − 478 to − 584 (**K–O**) and -785 to -874 (lower panel **P–T**) were analysed by 4 sets of primers in mouse stomach cells treated with RAN + lime for 0, 100 and 300 days. Chromatin was cross-linked, fragmented and immunoprecipitated with no antibody (as a negative control), with Histone 3 antibody (as a positive control) or anti-H3K4me3, H3K9ac, H318ac and H3K9me3 ChIP-grade antibodies. The purified DNA was used to amplify with four sets of primer pairs covering four regions (+ 528 to + 626 with Primer 1; − 83 to − 192 with Primer 2; − 478 to − 584 with Primer 3 and − 785 to − 874 with Primer 4) of the securin promoter by qPCR. As input, 10% diluted chromatin fragments were retained and used in qPCR for the enrichment analysis. The percentage of input values represents the mean of four different animals ± SEM. Data were analysed using one-way ANOVA with Tukey’s multiple comparison post-tests. P values less than 0.05 are considered significant

**Fig. 5:**
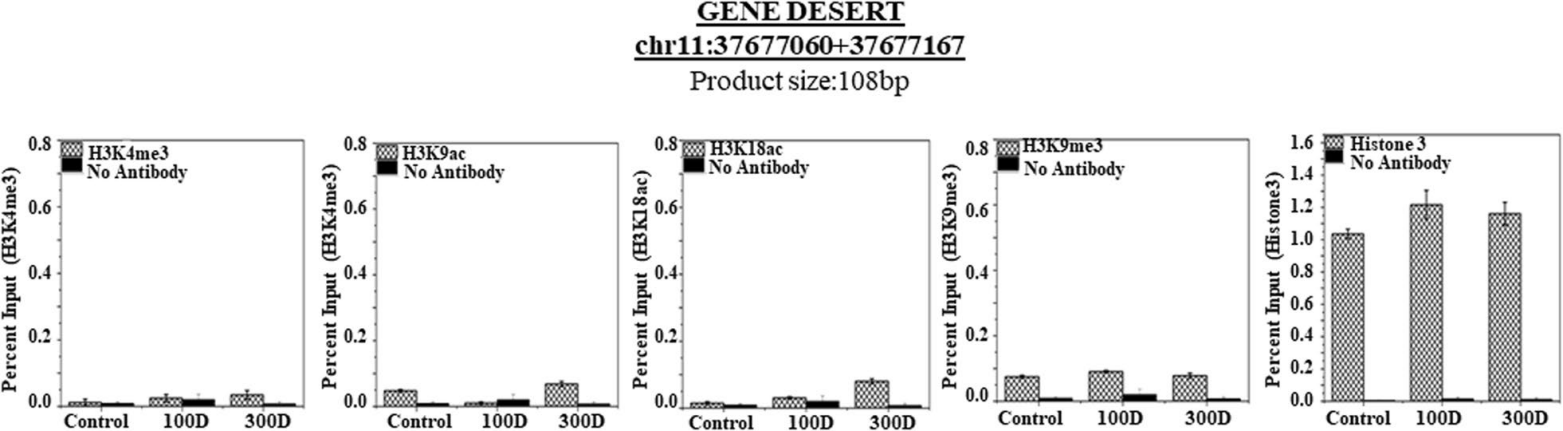
ChIP analysis of histone H3 methylation and acetylation at the gene desert region of chromosome 11 in mice stomach cells. ChIP-qRT-PCR assays for H3K4me3, H3K9ac, H3K18ac and H3K9me3 recruitment in the gene desert regions were analysed and served as negative control

To determine the recruitment of posttranslational modifications of histone H3 (methylation/ acetylation of histone H3 lysine 4, 9 and 18) in the promoter region of the securin gene, a chromatin immunoprecipitation (ChIP) assay was performed on stomach tissues in untreated and 100- and 300-days of RAN + lime-treated samples. The assays were then analyzed by qPCR with four primers targeting the regions representing upstream to downstream of the promoter of the securin gene. Figure 4 shows the results of ChIP experiments (enrichment of histone marks on the securin promoter indicated in % input). The data show that RAN + lime treatment led to a significant increase in the levels of H3K4me3, H3K9ac and H3K18ac in 300-day treated samples with respect to untreated one with the primer 2 (Fig. 4F–H), primer 3 (Fig. 4K–M) and primer 4 (Fig. 4P–R). Interestingly, the level of H3K9me3 was reduced significantly in 100- and 300-day-treated samples compared to the untreated control with these 3 sets of primers (Fig. 4I,N,S). It is also noted that the degree of increase in the level of H3K4me3, H3K9ac after 300 days treatment was not significant with the primer 1 and the level of H3K18ac did not show any change. The results indicate that the quantity of DNA fragments retrieved from the immunoprecipitated samples was maximum in the -83 to -192 region than further upstream of the promoter for H3K4me3, H3K9ac, and H3K18ac. ChIP-assay was also performed in the gene desert region of chromosome 11, as a negative control, to determine the recruitment of posttranslational modifications of histone H3 before and after RAN treatment. The data indicate that the amount of DNA fragments retrieved from the immunoprecipitated samples were negligible in both control and treated samples (Fig. 5).

**Fig. 6:**
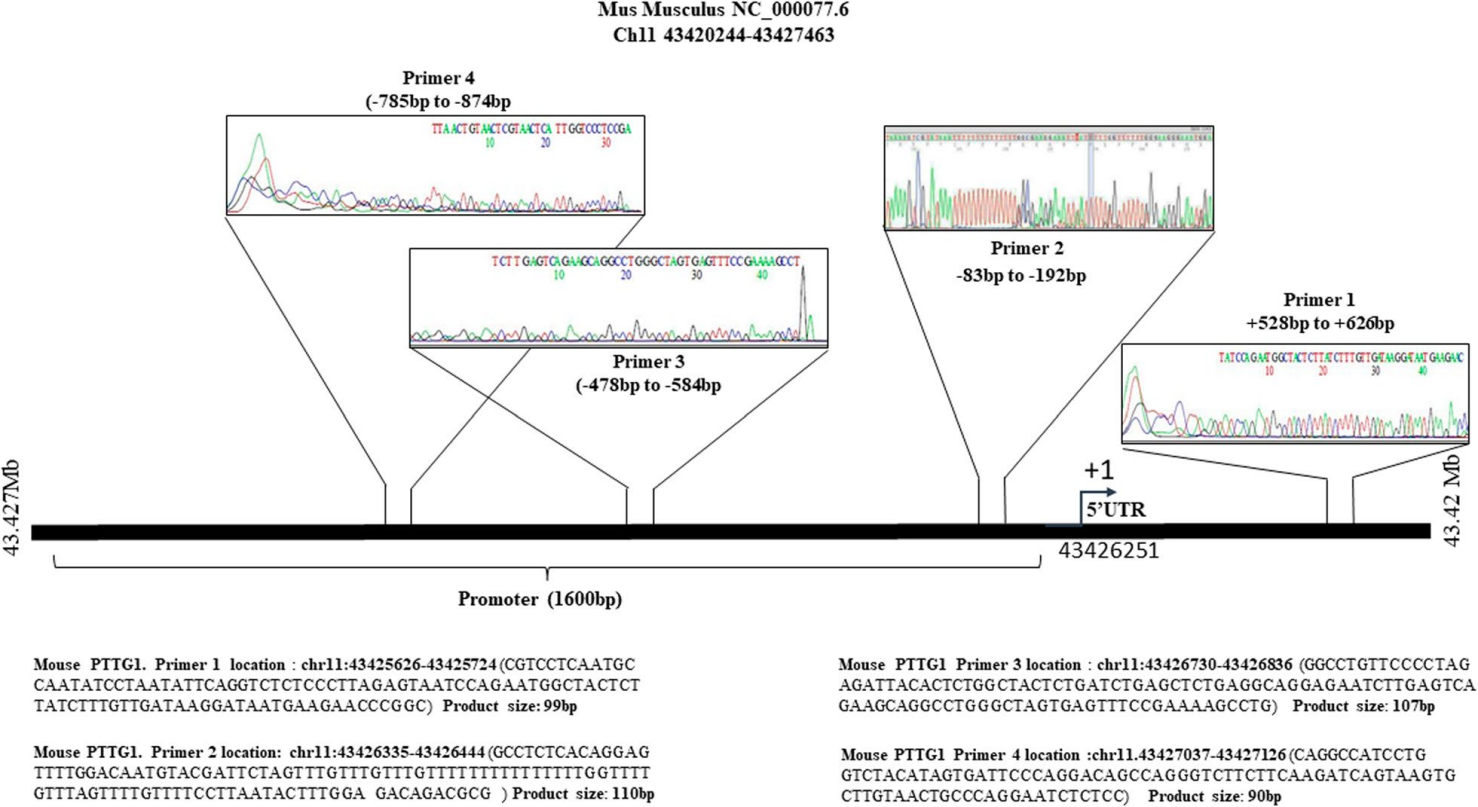
Sequencing of qRT-PCR products: Schematic diagram depicting the position of the qRT-PCR product that was sequenced and matched in the promoter region (− 478 to − 584 amplified by Primer 1; and − 785 to − 874 amplified by Primer 2) of the securin gene in mice. The transcription initiation site (+ 1) is indicated with an arrow. The sequence details of both amplified products are shown below

The product size of the qPCR was 99, 110, 107 and 90 bp for the primer 1, 2, 3 and 4, respectively. All these products from the promoter region were sequenced and then blasted (NCBI nucleotide blast) with the mouse genomic sequences. It was matched with the mouse PTTG1/securin gene present on chromosome 11 (Fig. 6). Primer 2 was designed in the core promoter region contains a stretch of T-rich region and the maximum quantity of DNA fragments was retrieved from the immunoprecipitated samples at this core promoter region.

### Gene expression profiles of histone modifying enzymes after RAN treatment

**Fig. 7:**
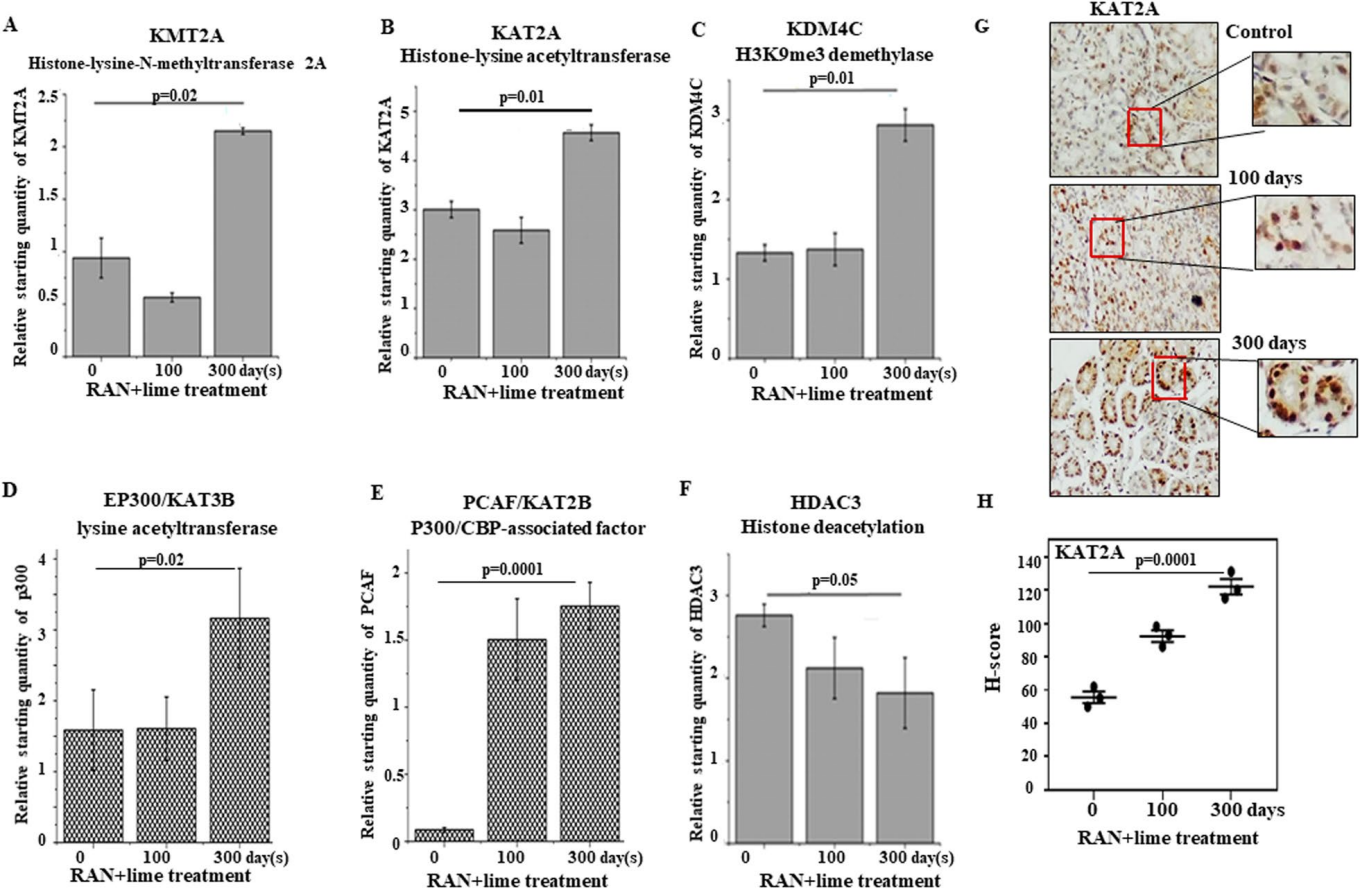
Expression of epigenetic chromatin modification enzymes after RAN treatment. Stomach cells of mice treated with RAN + lime for 0, 100 and 300 days and analyzed by qRT-PCR for **A** histone-lysine-N-methyltransferase 2A (KMT2A), **B** histone acetyltransferase (KAT2A), **C** demethylase (KDM4C), **D** histone deacetylation (HDAC3), **E** EP-300/KAT3B and **F** PCAF/KAT2B gene expression. Data are the mean ± SEM of a representative experiment performed in untreated control (N = 4) and treated (N = 4 in each category). The values are normalized to the respective GAPDH values. Data were analysed using one-way ANOVA with Tukey’s multiple comparison post-tests. P values are shown in all and considered significant when the values are less than 0.05. **G** Representative immunohistochemical staining (40x) of mouse stomach shows that Histone-lysine acetyltransferase (KAT2A) positive cells increase with the treatment duration. Magnification (100x) of the marked area is shown. **H** Scatterplot of H-scores based on IHC for KAT2A-positive cells. The magnification of all these images is 40x. Magnification (100x) of the marked area is shown

We have shown that RAN exposure increases the expression of the securin in stomach cells by increasing H3K4me3 and both H3K9 and H3K18 acetylation in the promoter region of securin. The present qRT-PCR data show that lysine methyltransferase 2A (KMT2A), lysine acetyltransferase 2A (KAT2A), lysine acetyltransferase 3B (EP300) and P300/CBP-associated factor (PCAF) is increased significantly after 300 days of feeding with RAN + lime (Fig. 7A–E). The expression pattern of a histone demethylase (KDM4C), which specifically targets tri- and dimethylated lysine 9 of histone H3 (H3-K9me3 and me2), was analyzed and significant upregulation was observed after 300 days of feeding (Fig. 7C). Consistent with KAT2A and KAT3B upregulation, the expression of histone deacetylase 3 (HDAC) was downregulated (Fig. 7F). Further, immunohistochemical staining on serial sections of mouse stomach showed an increase in the level of lysine acetyltransferase 2A (KAT2A) after RAN-exposure (Fig. 7G). The scatterplot of H-scores based on IHC for KAT2A-positive cells showed in Fig. 7H. We have shown that RAN exposure alters the gene expression pattern of chromatin modification enzymes, thereby affecting the dynamics of histone modifications that regulate transcriptional control in vivo in mouse stomach cells.

## Discussion

It is known that an increased level of securin facilitates genome instability, which is associated with a subset of genomic rearrangements leading to a worse prognosis in a broad range of cancerous tissues [17, 18]. Extensive research on this gene has been performed due to its clinical importance, however, the precise mechanisms by which securin induces its oncogenic function and the mechanisms that regulate its overexpression remain unknown. This study was carried out with an aim to delineate the molecular mechanism underlining securin upregulation after RAN exposure in mouse system since its exposure to RAN was regulated in a proper manner. This is not possible in humans due to interference with several other compounding factors (tobacco chewing or smoking, alcohol consumption and various types of non-vegeterian foods) that led to confusion. Here, the mice were treated with RAN extract with lime whose dose was increased periodically to mimic the human consumption style where the dose is increased gradually. Studies in different animal species have shown tumour induction in oesophageal and stomach tissues by RAN extract, which was administered by different means, such as oral intubation [38], mixing with the diet [39] or drinking water [6]. In the present study, the mouse stomach was considered because of its greater exposure after ad libitum administration of RAN extract with lime in drinking water. Since the initial response of the cells after RAN-exposure is similar both in mice and humans such as, induction of precocious anaphase and Securin upregulation (6,7), therefore the present mouse result can be extrapolated on human subjects.

In order to evaluate the mechanism of securin upregulation, a sequencing scan in several human pituitary adenoma biopsies were performed and failed to identify any promoter mutation of the securin gene. Therefore, it is believed that promoter mutation does not play any role in its enhanced expression [40, 41]. Furthermore, no loss of heterozygosity has been reported for the region mapping the PTTG1/securin locus [41]. On the other hand, it was demonstrated that the exposure with RAN-alkaloids creates more relaxed structure of chromatin in mouse system which could be a causative factor for transcriptional enhancement of key gene(s) relevant to carcinogenesis [21]. Therefore, the analysis of histone covalent modification patterns both at the global level and in the promoter region of the securin gene is worth pursuing because such modifications do have profound effects on gene promoter activity and have been extensively linked to cancer [42]. However, the methylation status in a CpG island at the proximal promoter region of the PTTG1/securin gene was not evaluated in this study since no methylation was observed in either healthy tissues or differentiated thyroid carcinoma samples, or in prostate cancer cell lines regardless of the expression status of PTTG1/securin gene [41].

The present results show hyperphosphorylation of Rb and upregulation of E2F1 in RAN treated samples; therefore, such deregulation of the Rb-E2F1 circuit might be involved in the upregulation of securin and its oncogenic role. Although pRB mutations are rare in oral cancer, however, its regulatory circuit is abrogated [43]. Such, altered expression of Rb and E2F was noted to be associated not only with the clinically aggressive oral cancers but also after tobacco/betal quid use [44]. Earlier, it was demonstrated that the expression of hPTTG1 is regulated by the Rb/E2F1 pathway [29], which plays an important role in carcinogenesis [45, 46]. In pituitary tumors, it has been reported that E2F1 affects PTTG1 expression [47]. Moreover, microarray analyses identified securin and several other mitotic checkpoint proteins, such as Bub1, Bub3 and Cdc20, as putative E2F1 targets [48].

Analysis of posttranslational histone H3 modifications both at the global level and in the promoter region of the securin gene was performed after RAN exposure. A significant increase in trimethylation at the H3K4 residue and acetylation at the H3K9 and H3K18 residues in stomach tissue were noted. Trimethylation of H3 lysine 4 is strongly associated with transcriptional activation and such epigenetic modification is usually observed near transcriptional start sites of highly expressed genes [49]. H3K4me3 is considered a well-established marker of active gene promoters [50], and enzyme complexes involved in such modifications have been implicated in oncogenesis [51, 52]. In addition, changes in acetylation signalling due to misregulated HATs or HDACs can cause chromatin decompaction, which leads to abnormal gene expression, including activation of proto-oncogenes [53], and impair DNA damage responses [54], which together can impact genome-epigenome stability.

The present ChIP-qPCR data have revealed that the quantity of DNA fragments retrieved from the immunoprecipitated samples was maximum in the − 83 to − 192 region than further upstream of the promoter for H3K4me3, H3K9ac, H3K18ac and H3K9me3. It indicates that a maximum elevation of hypermethylation of H3K4 and hyperacetylation of H3K9 and H3K18 within the core promoter region of securin gene and the present data support the previous observation that − 313 to − 150 bp are critical for promoter activity in mouse PTTG1/securin gene [34]. Increased H3K4me3 in the promoter is functionally correlated with an increase in acetylation of H3K9, and both histone modifications are considered to be a major modification of transcribed genes [55, 56]. Moreover, transcription facilitator H3K9 acetylation is mutually exclusive to transcriptionally repressive H3K9 methylation [57]. Interestingly, the present data show that a reduced level of H3K9me3 corresponds to an increase in H3K9ac levels, indicating that the cross-regulation between H3K9 methylation and H3K9 acetylation plays a role in establishing a transcriptionally active state of the securin promoter in response to RAN-mediated stimulation.

The present posttranslational modifications of histones are catalyzed by a number of enzymes, and the present data clearly show higher expression of lysine-N-methyltransferase 2A (KMT2A) and lysine acetyltransferase (KAT2A) and decreased levels of HDAC3 after RAN exposure, which supports the observed elevation in H3K4me3, H3K9ac and H3K18ac in the promoter region of the securin gene. In addition, the present results show an enhanced level of p300 and P300/CBP‐associating factor (PCAF) after 300 days of RAN feeding. EP300, also known as KAT3B, is a large, multidomain protein that, in addition to its catalytic HAT domain, contains bromodomains that bind acetylated histones and are required for chromatin binding for transcriptional activation [58]. PCAF is a histone acetyltransferase that primarily acetylates H3 histones and has a strong association with tumor initiation and progression [59]. It has been demonstrated that PCAF is specifically required, whereas p300 is involved in H3K18ac in cells [60]. Therefore, higher expression of enzymes regulating methylation and acetylation of histones in the stomach cells of mice after RAN exposure indicates its association with gastric cancer. Similar higher expression of enzymes regulating methylation and acetylation of histones in esophageal cancer of northeastern population of India having the habit of areca nut chewing was demonstrated [25]. They had also performed tissue microarray in an independent cohort of 75 patients revealed higher nuclear protein expression of KAT8 and PRMT1 in tumor similar to mRNA expression. In this study, increased level of lysine acetyltransferase 2A (KAT2A) after RAN-exposure was demonstrated in the stomach cells. Thus, it is believed that the present observed higher mRNA levels of these enzymes give higher levels of respective proteins in the cells.

It is interesting to note that RAN exposure hyper-phosphorylates Rb and thus allows E2F1 to drive transcription of its target genes, including PTTG1/securin. Although, the pRB/E2F pathway has been extensively studied for decades, its involvement in other noncanonical functions is emerging through its interaction with chromatin modifier proteins [61]. Therefore, it might be possible that the present histone3 acetylation at K9 and K18 could be facilitated by inactivated pRb, as demonstrated in DNA break repair [62]. In addition, pRb also regulates H3K4 methylation by inhibiting the demethylase action of KDM5A through its interaction [63]. Thus, in a normal cell pRb, a putative tumor suppressor protein, not only represses transcription by inhibiting the E2F family of transcriptional factors but also plays a pivotal role in the maintenance of chromosome structure and stability via physical interactions with chromatin-related proteins to silence transcription [64]. It seems that RAN exposure leads to pRb hyperphosphorylation, which subsequently develops chromosomal instability (CIN) and aneuploidy as reported earlier [65]. It has been demonstrated that induction of CIN and overexpression of securin are significantly associated with gastric cancer in mice [6] and both oral and esophageal cancers in humans with RAN consumption habits [7]. Moreover, association of Securin/PTTG1 upregulation and gastric cancer in human was also demonstrated [66]. Therefore, the results of the present study have led to the hypothesis that securin overexpression and subsequent elevation of CIN and aneuploidy are probably byproducts of inactivation of the pRB pathway. Support for this idea comes from several earlier studies that have shown the expression signature of more than 10 genes, including PTTG1/securin, that show a strong correlation with CIN are putative E2F targets, and are surrogate markers of pRb inactivation [67, 68].

## Conclusions

This study shows that overexpression of securin after RAN exposure could be due to deregulation of the Rb-E2F1 circuit and subsequent elevation of H3K4me3, H3K9ac and H3K18ac in the promoter region of the securin gene. However, RAN-mediated deregulation of E2F cell cycle targets, including securin, concurrent with loss of genome stability hindered the ability to assess whether pRB could maintain genome integrity independent of E2F transcriptional control. Further in-depth research is required to understand how many cellular processes are altered when RB1 is mutated. Expanding our understanding of how epigenetic modifications contribute to oral, esophageal and gastric cancers in RAN users may not only improve the knowledge of pathogenesis but also provide new novel biomarkers for diagnosis or disease outcome prediction and/or response to therapy.

## Supplementary Information

**Additional file 1.** Experimental Procedures. **Table S1.** Mice qRT-PCR primers used. **Fig. S1.** Immunohistochemical (IHC) staining for histone H3 in mice stomach cells.

## Data Availability

All data generated or analysed during this study are included in this published article.

## Abbreviations

RAN: Raw areca-nut
PTTG1: Pituitary tumor transforming gene 1
CIN: Chromosomal instability
pRb: Protein retinoblastoma
H3K4me3: Histone H3 lysine 4 trimethylation
H3K9ac: Histone H3 lysine 9 acetylation
ChIP: Chromatin immunoprecipitation
KMT2A: Lysine methyltransferase 2A
KAT2A: Lysine acetyltransferase 2A
KDM4C: Histone demethylase
HDAC3: Histone deacetylase 3
PCAF: P300/CBP‐associating factor
