# Supplementary material for: Securin overexpression correlates with the activated Rb/E2F1 pathway and histone H3 epigenetic modifications in raw areca nut-induced carcinogenesis in mice
Source: Cancer Cell Int. 2022 Jan 15;22:30. doi: 10.1186/s12935-022-02442-z (PMC8761315; doi:10.1186/s12935-022-02442-z)
Supplement: Supplementary file 1 — Additional file 1. Experimental Procedures. Table S1. Mice qRT-PCR primers used. Fig. S1. Immunohistochemical (IHC) staining for histone H3 in mice stomach cells. [file 12935_2022_2442_MOESM1_ESM.docx]

**Additional file 1 Section**

**Securin overexpression correlates with the activated Rb/E2F1 pathway and histone H3 epigenetic modifications in raw areca nut-induced carcinogenesis in mice**

**Nabamita Boruah, Chongtham Sovachandra Singh, Pooja Swargiary, Hughbert Dkhar^1^, Anupam Chatterjee^#^**

Molecular Genetics Laboratory, Department of Biotechnology & Bioinformatics, North-Eastern Hill University, Shillong, Meghalaya, India-793022;

**^1^**Histopathology Division, Nazareth Hospital, Laitumkhrah, Shillong-793003, India; Nazareth Hospital, Laitumkhrah, Shillong-793003, India.

**^#^Corresponding Author**: Anupam Chatterjee, Department of Biotechnology & Bioinformatics, North-Eastern Hill University, Shillong, Meghalaya-793022, India.

Tel: +91 364 2722403; Email: [anupamchatterjee@nehu.ac.in](mailto:anupamchatterjee@nehu.ac.in); [chatterjeeanupam@hotmail.com](mailto:chatterjeeanupam@hotmail.com)

**Additional file 1: Experimental Procedures**

**Immunohistochemical analysis**

IHC staining was performed on formalin fixed, paraffin embedded tissues that were sectioned at 5 µm thickness. Slides were deparaffinized, rehydrated and treated with 3% H_2_O_2_ in PBS. Antigen retrieval was done with 0.01% sodium-citrate buffer followed by blocking in PBST containing 0.1% BSA and 10% FBS. Primary antibody incubation was carried out for overnight. Slides were washed and incubated with appropriate biotinylated secondary antibody for 1 hr at room temperature. Following washing slides were treated with streptavidin-HRP (1:1000) and subsequently washed (PBS containing 0.1% Tween-20) and color was developed with DAB+ H_2_O_2_. Slides were counterstained with haematoxylin, washed and mounted in DPX (Sigma-Aldrich, USA). The staining intensity of the images was evaluated by two independent investigators.

In the regions of the histologic sections, the chromogenic immunolabeling was systematically categorized into four groups: 0 (no labelling), 1+ (weak labelling), 2+ (moderate labelling), and 3+ (strong staining; observable with 10x objective). Semi-quantitative staining analysis was done by H-score by counting 500 cells from ten different fields in the slides considering low, moderate and higher intensity of Securin~~e~~ and H3K4me3, H3K9ac and H3K9me3 expression. The percentage of positive cells with a given intensity for each sample was determined independently by a pathologist and a trained reader. A single manual H-score based on a scale of 0 to 250 was generated for each labelled section by taking the sum of the percentage of cells labelling 1+, double the percentage of cells labelling 2+, and triple the percentage of cells labelling 3+ (H-Score = ((%3+) × 3) + ((%2+) × 2) + (%1+)). Student’s t-test was performed for comparing the expression of these two genes in untreated samples with the samples treated with RAN+lime. Statistical significance was considered when p-value is less than 0.05.

**Chromatin immunoprecipitation and ChIP- qRT-PCR**

Scraped and minced cells from stomach tissue of mice (20mg for each ChIP reaction) were crosslinked with 1% formaldehyde for 20 min at room temperature, then quenched by 0.125M glycine and incubated for 5 min at room temperature. The cells were homogenized (15-20 strokes with Dounce homogenizer) in homogenization Buffer (10 Mm HEPES at Ph 7.9, 0.5% NP-40, 1.5 Mm MgCl2, 10 Mm KCl, 0.5 Mm DTT, 1Mm PMSF and 1X PIC (Protease inhibitor cocktail- 1 Mm PMSF, 1 µg/ml aprotinin and 1 µg/ml pepstatin A) on ice (1 ml per 200 mg tissue). After centrifugation at ~960´g for 5 min at 4 °C, supernatant was removed. Cell pellets were lysed in nuclei lysis buffer (50Mm Tris-HCl (Ph 8.0), 10 Mm EDTA, 1% EDTA, 1X PMSF and 1X PIC (protease inhibitor cocktail-1 Mm PMSF, 1 µg/ml aprotinin and 1 µg/ml pepstatin A; Sigma P8340; USA) and sonicated (Sonicator UP100h Hielscher, 15-20 cycles, 30 Sec ON and 60 Sec OFF. Amplitude:60%) with Shearing buffer (50Mm Tris-HCl (Ph 8.0), 10 Mm EDTA, 1% EDTA, 1X PMSF and 1X PIC) on ice (100Ml/20mg tissue). After centrifugation at ~21,000´ g for 10 min at 4 °C, supernatant was collected. The supernatant was diluted with an equal volume of ChIP dilution buffer (1.1% Triton X-100, 1.2Mm EDTA, 16.7Mm tris-HCl (Ph 8.0), 167Mm NaCl, PMSF and PIC) and distributed equally for each antibody. 10% of diluted chromatin kept as Input. Immunoprecipitation was performed with antibodies specific to H3K4me3 (ab8580), H3K9ac (ab12179), H3K9me3 (ab8898), H3K18ac(ab1191) and Histone 3 (ab1791) with protein A/G beads (Pierce™ Protein A/G Agarose, Cat no. 20421) incubated for 7-8 hours at 4°C on orbital rotor while only protein A/G beads with cell lysate without antibody kept as control in the same process. After incubation, precipitates were sequentially washed with Low Salt Buffer (150Mm NaCl, 0.1% SDS, 1% Triton X-100, 2Mm EDTA, 20Mm Tris-HCl (Ph 8.0)), High Salt Buffer (500Mm NaCl, 0.1% SDS, 1% Triton X-100, 2Mm EDTA, 20Mm Tris-HCl (Ph 8.0)), LiCl buffer (0.25 M LiCl, 1% NP-40, 1% sodium deoxycholate, 1% Mm EDTA, 10Mm Tris-HCl (Ph 8.0)) and 1X TE (10Mm Tris-HCl, Ph 8.0, 1 Mm EDTA). DNA was then eluted with 1% SDS and 0.1 M NaHCO_3_. The eluates together with 5M NaCl incubated for at least 6 hours to overnight at 65°C to reverse the formaldehyde cross-linking. Next day, RNase (1mg/ml) added to eluates incubated at 37°C for half an hour followed by 1 M Tris-Cl, 0.5M EDTA and Proteinase k (20mg/ml) incubation at 45°C for 1.5 to 2 hours. Chloroform in the equal volume added to the above contents to remove remaining proteins and further ethanol and salt purification of the aqueous phase was done to precipitate the pure DNA. DNA fragments were then dissolved in 1X TE and stored at -20°C. Purified DNA was analysed using Quantitative Real Time PCR using reagents containing SYBR green: ΔΔCt method (BioRad CFX system) using primer for Mouse PTTG1 promoter designed from UCSC browser and Primer 3 software (Version 0.4.0). Real Time PCR products were purified using SIGMA PCR clean up kit (NA 1020) and submitted for sequencing to Agrigenome, Kochi, India.

**RNA extraction and qRT-PCR**

Cells were collected from the inner layer of stomach of mice from untreated and treated for 100- and 300-day with RAN+lime (n=4 in each category). Total RNA was isolated with Trizol using the RNeasy Mini Kit (Qiagen Co. Limburg, Netherlands) according to the manufacturer’s protocol. From 1μg of total RNA, cDNA synthesis was performed using QuantiTect Reverse Transcription kit (Qiagen GmbH, Hilden, Germany) according to the manufacturer’s protocol. qRT-PCR was performed using in 96-well optical reaction plates in a Bio-Rad CFX96 Real-Time PCR Detection System. The qRT-PCR reactions were prepared using SYBR® Select Master Mix (Life Technologies), and the following conditions were used: 95°C for 5 min, 40 cycles of 95°C for 30s, 60°C for 30s and 72^o^C for 30s. The primers of target genes used for this analysis were KMT2A, KAT2A, HDAC3, KDM4C, KAT3B, KAT2B and GAPDH was used as the reference gene. The gene copy numbers of KMT2A, KAT2A, HDAC3, KDM4C, KAT3B and KAT2B were calculated by using a relative standard curve that was constructed using QIAGEN Mouse Xpress Ref Universal Total RNA (Cat no. 338114). Amount of target (Genes of Interest-GOI) and endogenous reference (GAPDH) were determined from the appropriate standard curve of mouse universal RNA. Quantities of GOI were normalized with endogenous reference.

**Table S1. Mice qRT-PCR primers used**

**Target name Primer Sequence Product size**

**KMT2A**  F-5`-CGATGACAACCGACAGTGTGCA-3` 123 bp

R-5`-GCTGACCACAAAGCACAGTTCAC-3`

**HDAC3** F-5`-AATGTGCCCTTACGAGATGG-3` 237 bp

R-5`-GTAGCCACCACCTCCCAGTA-3`

**KAT2A**  F-5`-GAAGAGGACCCTCATCCTCA-3` 273 bp

R-5`-GGAGAATTTGCCCCGTAGAT-3`

**KDM4C** F-5`-GATGACTGGCCTTACGTGGT-3` 249 bp

R-5`-CTTCACACAGTTTCGGCTCA-3`

**P300/KAT3B** F-5’**-** CTCCGGATCCTGCTGCTTTA-3’ 72 bp

R-5’- CCCCTTCCACTTTACGAGCAT-3’

**PCAF/KAT2B** F-5’-TGTCATTGGTGGTATCTGT-3’ 166 bp

R-5’-ATATGTGAGGAAGTTGAGGAT-3’

**Additional file 1 Images**

**Figure legends**

**Fig. S1: Immunohistochemical (IHC) staining for histone H3 in mice stomach cells.** Representative images show the level of histone H3 positive cells in the untreated and treated mice **A**. in the cells of stomach. The level of histone H3 positive in untreated and treated mice analyzed by H-scores and were shown as the mean H-score ± SEM in **B.** The magnification of all these images is x40.

**Stomach tissues**

**A**


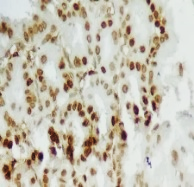


**Untreated**

**control**


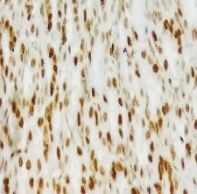


**100 days**


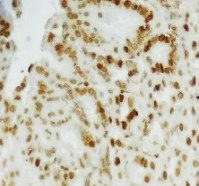


**300 days**

**B**


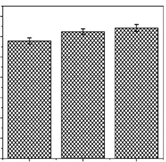


**140**

**120**

**100**

**80**

**60**

**40**

**20**

**0**

**Mean H-score of Histone 3**

**Treatment with RAN+lime**

**0 100 300 day(s)**

**Fig. S1**
